# Association of Polymorphisms in Inflammatory Cytokines Encoding Genes With Anti-N-methyl-D-Aspartate Receptor Encephalitis in the Southern Han Chinese

**DOI:** 10.3389/fneur.2020.553355

**Published:** 2020-12-11

**Authors:** Xing Li, Jiajia Zhu, Yu Peng, Hongbing Guan, Jinyu Chen, Zhanhang Wang, Dong Zheng, Nan Cheng, Honghao Wang

**Affiliations:** ^1^Department of Neurology, Guangzhou First People's Hospital, School of Medicine, South China University of Technology, Guangzhou, China; ^2^Department of Neurology, Nanfang Hospital, Southern Medical University, Guangzhou, China; ^3^Key Laboratory of Oral Medicine, Guangzhou Institute of Oral Disease, Affiliated Stomatology Hospital of Guangzhou Medical University, Guangzhou, China; ^4^Department of Neurology, Guangdong 999 Brain Hospital, Guangzhou, China; ^5^Department of Neurology, Brain Hospital Affiliated to Guangzhou Medical University, Guangzhou, China; ^6^Hospital Affiliated to Institute of Neurology, Anhui University of Traditional Chinese Medicine, Hefei, China

**Keywords:** anti-NMDAR encephalitis, inflammatory cytokines, single nucleotide polymorphisms, IL-1β, Southern Han Chinese

## Abstract

**Background:** Single nucleotide polymorphisms (SNPs) that occur within genes encoding inflammatory cytokines can result in quantitative or qualitative changes in their expression or functionality, potentially leading to the development of anti-N-methyl-D-aspartate receptor (NMDAR) encephalitis. This study sought to evaluate the relationship between SNPs in inflammatory cytokines genes and the incidence of anti-NMDAR encephalitis in the Southern Han Chinese.

**Methods:** In total, we enrolled 107 patients with anti-NMDAR encephalitis as well as 202 inpatient controls who had no first-degree relative with autoimmune diseases. Genotyping determination of all 309 patients was conducted for the IL-1β rs16944, IL-4 rs2243250, IL-4 rs2070874, IL-6 rs1800796, IL-10 rs1800872, and IL-17 rs2275913 gene SNPs.

**Results:** We observed statistically significant differences in the frequencies of G allele in IL-1β rs16944 between anti-NMDAR encephalitis and controls (*p* = 0.017). Also, IL-1β, IL-4, IL-6, IL-10, and IL-17 SNPs were not associated with the disease (*p* > 0.05).

**Conclusions:** We found that patients with anti-NMDAR encephalitis exhibit a distinct immunological profile, and we found that the decreased frequency of G allele in IL-1β rs16944 showed a protective role for anti-NMDAR encephalitis in the Southern Han Chinese.

## Introduction

Anti-N-methyl-D-aspartate receptor (anti-NMDAR) encephalitis is a form of encephalitis affecting the central nervous system (CNS) that has only recently been characterized as a form of autoimmune disease (1). The specific mechanisms of the pathogenesis are still yet to be fully illuminated.

Anti-NMDAR encephalitis is thought to be driven by pathogenic immune- and inflammation-mediated activation within the CNS, with both T and B cells functioning to drive disease-related immunopathogenesis (2). Liba et al. showed remarkable changes of chemokines levels related to B cells and T cells, such as tumor necrosis factor-α (TNF-α), interleukin-17A (IL-17A), and CXCL13 in the cerebrospinal fluid (CSF) at the early stage of anti-NMDAR encephalitis (3). We have previously demonstrated that anti-NMDAR encephalitis is associated with a significant increase in levels of IL-1β, IL-6, and IL-17A in the CSF relative to control patients (4). IL-10 and IL-4, are two cytokines that have been found to have strong links to encephalitis (5).

Host genetic determinants, apart from environmental elements, are also major causes of susceptibility to or consequence of antoimmune encephalitis (6). Single nucleotide polymorphisms (SNPs) arising within the promoter or protein-coding regions of genes that code for inflammatory cytokines can alter their expression or functionality (7). It has been indicated that certain SNPs encoding cytokines in the genes could not only increase susceptibility to some antoimmune encephalitis but also change the prediction and course of disease (8–10). Given that these inflammatory cytokines can serve as key drivers of autoimmune encephalitis, polymorphisms in genes such as *IL1*β (11), *IL4* (12), *TNFA* (13), and *IL10* (14) are thought to be potential host susceptibility factors associated with anti-NMDAR encephalitis and other autoimmune disorders. Nevertheless, recent one study found that GRIN1 polymorphism do not affect susceptibility in anti-NMDAR encephalitis (15). As such, the present study sought to evaluate the relationship between SNPs in inflammatory cytokine genes and the incidence of anti-NMDAR encephalitis among a Southern Chinese population of Han ethnicity.

## Methods

### Study Design

Herein, we conducted a case-control study of patients presenting to the Department of Neurology of Nanfang Hospital from March 2017 to February 2019. In total, 107 patients with anti-NMDAR encephalitis were included in the study, all fulfilling the diagnostic criteria described in Day et al. (16). Anti-NMDAR encephalitis diagnoses were confirmed based on patient symptoms, cell-based assays, and via the detection of anti-NMDAR within the CSF of affected patients. In addition, 202 consenting volunteers, having no first-degree relative with autoimmune diseases, were recruited as selected controls. All the subjects were born in Southern China and Southern Han Chinese.

### SNPs Genotyping

Peripheral blood samples were collected and stored at −80°C. DNA was extracted from serum samples collected from each patient using a DNA purification kit (Roche, Germany) according to provided directions. After extraction, genotype was carried out for polymorphisms determination in IL-1β rs16944, IL-4 rs2243250, IL-4 rs2070874, IL-6 rs1800796, IL-10 rs1800872, and IL-17 rs2275913 using TaqMan commercial probes (Applied Biosystems, CA, USA). After standardization of the PCR conditions, sequencing was carried out using an automated ABI Prism 3700 DNA sequencer (Applied Biosystems, Foster City, CA). Genotyping was deemed successful if the concordance rate between duplicates was ≥95%. For samples that did not give a clear genotype, the PCR and sequencing was repeated until the results were unequivocal. For details pertaining to SNPs analyzed in the present study, see Table 1. All RT-PCR reactions were conducted using 12 μl of TaqMan SNP genotyping master mix (Life Technology, CA, USA), 6 μl of DNA, 2.8 μl dH_2_O, and 0.6 μl of probes. Thermocycler settings were: 95°C for 8 min, 60 cycles of 95°C for 10 s, and 40°C for 2 min.

**Table 1:** Genotype and allele frequencies of IL-1β, IL-4, IL-6, IL-10, and IL-17 in anti-NMDAR encephalitis and control groups.

**Gene and genotype/allele**	**Anti-NMDAR encephalitis (%) *n* = 107**	**Controls (%)*n* = 202**	**Crude *p*-value**	**OR (95% CI)**	**Adjusted *p*-value**
IL-1β; rs16944					
AA	34 (31.8)	42 (20.8)			
AG	43 (40.2)	85 (42.1)	0.075		
GG	30 (28.0)	75 (37.1)			
A	111 (51.9)	169 (41.8)	0.017^*^	1.499 (1.074–2.091)	0.034^*^
G	103 (48.1)	235 (58.2)			
IL-4; rs2243250					
CC	21 (19.6)	36 (17.8)			
CT	23 (21.5)	29 (14.4)	0.212		
TT	63 (58.9)	137 (67.8)			
C	65 (30.4)	101 (25.0)	0.152	1.309 (0.906–1.891)	0.304
T	149 (69.6)	303 (75.0)			
IL-4; rs2070874					
CC	14 (13.1)	31 (15.3)			
CT	15 (14.0)	32 (15.8)	0.754		
TT	78 (72.9)	139 (68.8)			
C	43 (20.1)	94 (23.3)	0.366	0.829 (0.552–1.245)	0.732
T	171 (79.9)	310 (76.7)			
IL-6; rs1800796					
CC	18 (17.3)	36 (17.8)			
CG	15 (14.4)	25 (12.4)	0.881		
GG	71 (68.3)	141 (69.8)			
C	51 (24.5)	97 (24.0)	0.889	1.028 (0.696–1.518)	1.778
G	157 (75.5)	307 (76.0)			
IL-10; rs1800872					
TT	41 (38.3)	81 (40.1)			
TG	44 (41.1)	78 (38.6)	0.912		
GG	22 (20.6)	43 (21.3)			
T	126 (58.9)	240 (59.4)	0.899	0.978 (0.698–1.370)	1.798
G	88 (41.1)	164 (40.6)			
IL17; rs2275913					
GG	35 (32.7)	70 (34.7)			
GA	41 (38.3)	71 (35.1)	0.858		
AA	31 (29.0)	61 (30.2)			
G	111 (51.9)	211 (52.2)	0.932	0.986 (0.707–1.373)	1.864
A	103 (48.1)	193 (47.8)			

* *p-value is significant*.

*OR, Odds ratio; CI, Confidence interval (calculated by logistic regression model)*.

### Statistical Analysis

SPSS 20.0 (IBM, IL, USA) was used for statistical testing. Genotype and allele frequencies were compared between patient groups using Fisher's exact tests and Pearson chi-squared tests. Relative risk was then assessed using odds ratios (ORs) and 95% confidence intervals (CIs), with *p* < 0.05 as the significance threshold.

## Results

### Participants

The mean ages for anti-NMDAR encephalitis and control cases were 29.84 and 27.88 years, respectively. In anti-NMDAR encephalitis 48 (44.9%) were male and 59 (55.1%) were female and in the control group 84 (41.6%) and 118 (58.4%) were male and female, respectively. Eight of anti-NMDAR encephalitis patients have teratomas.

### Genotyping

SNP allele and genotype frequencies assessed in IL-1β, IL-4, IL-6, IL-10, and IL-17 in the present study are compiled in Table 1. We did not identify any significant differences in SNP genotypes of IL-1β, IL-4, IL-6, IL-10, and IL-17 between anti-NMDAR encephalitis and control groups. In addition, we compared the frequencies of A and G alleles between the control and anti-NMDAR encephalitis groups. The results showed that the decreased frequency of alleles G in IL-1β rs16944 SNP has a protective effect on anti-NMDAR encephalitis (*p* = 0.017).

## Discussion

At present, pathogenesis mechanisms in anti-NMDAR encephalitis are being intensively studied. Analysis of SNPs in inflammatory cytokines encoding genes in a clinical context may be conducive to a better understanding of the course of disease. Our study was concentrated on the gene polymorphisms of cytokines related to T cell and B cell functions in anti-NMDAR encephalitis. In this case-control study, we studied the association of SNPs in IL-1β, IL-4, IL-6, IL-10, and IL-17 genes with anti-NMDAR encephalitis and controls in the Southern Han Chinese. We found that the frequencies of G allele of IL-1β rs16944 were notably decreased in anti-NMDAR encephalitis cohort compared with controls. The present study revealed an independent protective association between decreased frequency of G allele in IL-1β rs16944 and Anti-NMDAR encephalitis. To our knowledge, no other cytokine study in this disease has been published except the one by Leypoldt et al. (15).

Anti-NMDAR encephalitis is an autoimmune disease associated with a number of characteristic features such as neuronal loss, inflammation, and cellular infiltration (17). The exact factors that trigger autoimmune responses to NMDA receptors remain to be determined (18). However, IL-1β is thought to be capable of modulating blood-brain barrier permeability, the activation of glial cells, neuronal apoptosis and necrosis, inflammation, and immune cell infiltration within the CNS (19, 20). Our results and those of other studies further support a model wherein cells present within the blood can function as pro-inflammatory triggers from brain lesions in these patients, with initial increases in IL-1β occurring in blood cells. These initial increases in IL-1β expression contribute to blood-brain barrier breakdown and subsequent lesion development. Thus, IL-1β production by monocyte-derived macrophages and neutrophils is crucial for development of anti-NMDAR encephalitis.

Patients with anti-NMDAR encephalitis often exhibit activation of intrathecal IL-17 and IL-6 expression (21). Zeng et al. determined that more significant Th17 cell accumulation occurs within the CSF of anti-NMDAR encephalitis patients relative to healthy controls (22). In addition, anti-NMDAR encephalitis patients exhibit increased CSF levels of cytokines and chemokines such as IL-1β, IL-17, IL-6, and CXCL-13 relative to controls. Comparisons of genotypes between controls and those with anti-NMDAR encephalitis patients suggest that there are no significant differences in these groups with respect to analyzed SNPs in the IL-6 or IL-17 genes. A rational explanation for the discrepancies might be the different genetic background and the demographic differences of the studied populations that were shown to be very crucial in the variation of anti-NMDAR encephalitis course and severity.

In summary, the results of this study suggest that evaluating cytokine co-variation may offer additional insight into anti-NMDAR encephalitis pathophysiology and severity, especially in regard to different SNPs. The data obtained from this study demonstrated that the decreased frequency of G allele in IL-1β rs16944 might be acts as a protective factor against anti-NMDAR encephalitis.

## Data Availability Statement

The datasets presented in this study can be found in online repositories. The names of the repository/repositories and accession number(s) can be found in the article/Supplementary Material.

## Ethics Statement

The studies involving human participants were reviewed and approved by Medical Ethics Committee of Nanfang Hospital, Southern Medical University. The patients/participants provided their written informed consent to participate in this study. Written informed consent was obtained from the individual(s) for the publication of any potentially identifiable images or data included in this article.

## Author Contributions

HW conceived this study and designed the experiments. XL, JZ, YP, JC, and ZW collected the samples and clinical data. XL performed the experiments, analyzed the data, and wrote the manuscript. All authors read and approved the final version of the manuscript and agreed to submit it for publication.

## Conflict of Interest

The authors declare that the research was conducted in the absence of any commercial or financial relationships that could be construed as a potential conflict of interest.
